# Three-Dimensional Device-Free Localization for Vehicle

**DOI:** 10.3390/s20133775

**Published:** 2020-07-05

**Authors:** Manyi Wang, Jiaxing Yang, Binghua Huang, Yuan Yang, Yadong Xu

**Affiliations:** 1 The School of Mechanical Engineering, NanJing University of Science and Technology, NanJing 210094, China; manyi.wang@njust.edu.cn (M.W.); j.x.yang@njust.edu.cn (J.Y.); huangbinghua2018@njust.edu.cn (B.H.); 2 The School of Instrument Science and Engineering, Southeast University, NanJing 211189, China; yangyuan@seu.edu.cn

**Keywords:** device-free localization, three-dimensional, vehicle, wireless sensor network

## Abstract

Device-free localization (DFL) is a promising technique which could provide localization information for a target without requiring an electronic device. With the development of the smart city and smart transportation, DFL could form part of a basic technique that could be used to track and localize roadside vehicles. In this paper, some algorithms for three-dimensional (3D) DFL for vehicle surveillance are developed, including statistical methods for data, a method for communication link selection, a novel method of communication link weight allocation and some other minor approaches to obtain the location and approximate size of a static vehicle, as a basic technique of moving vehicle detection. Then, an experimental system is designed. Through security monitoring wireless sensor networks (WSN), real-time vehicle characteristics (i.e., location and size) are calculated automatically and intuitively displayed through a heat map. Experiments are performed to validate the effect of the proposal and the accuracy of the localization and size estimation.

## 1. Introduction

In recent years, the number of vehicles has increased rapidly, and traffic is universal in cities. Therefore, it is necessary to promote the development of the smart city and smart transportation to alleviate the traffic problem. Therefore, a real-time monitoring system is needed to identify and classify potential threats from a safe distance. Device-free localization (DFL) is an advanced technique that can realize positioning and tracking for a target only by analyzing the received signal strength (RSS) variance of wireless communication links introduced by the obstruction of the target, without the need for a device being equipped. It performs based on wireless sensor networks (WSN) formed by several wireless sensors. In recent related papers, DFL techniques have mostly been used for the two-dimensional localization of a small-scale target (e.g., people) [1,2,3,4,5,6,7]. Specifically, C.R. Anderson proposed a DFL method [8] for the scenario of roadside surveillance, offering a low-cost potential method to remotely monitor the situation of a vehicle with minimal investment of resources. Kassem and Jie also proposed two different methods to realize monitoring and speed estimation for a vehicle, respectively [9,10]. However, these methods are unable to obtain the scale of vehicle (i.e., length, height, and width). To this end, in order to developmore accurate roadside surveillance, the aim of this study is to obtain the location and scale of a vehicle simultaneously via DFL based on WSN.

The rest of the paper is organized as follows. Section 2 reviews the related works. Section 3 proposes a three-dimensional DFL method for roadside vehicles. Section 4 presents a selecting method for the obscured communication links based on the kernel distance. Section 5 describes how to allocate the weights of communication links and obtain a heat map and subsequently how to estimate the location and 3D characteristics of a vehicle. Section 6 validates the proposed model and system with extensive experiments. Finally, we present our conclusion in Section 7.

## 2. Related Works

DFL was first proposed by Youssef [11,12]; ever since, the field has attracted a great deal of research interest. Wilson et al. [1] proposed a radio tomographic image (RTI) model based on RSS shadow fading that utilizes the RSS difference between the online phase and offline phase to locate a target. In their later work, Wilson [13] utilized the online variance of RSS to improve the localization accuracy in the monitoring area with wind interference. In order to locate a moving target, Yang Zhao et al. [14] adopted a kernel distance method to calculate the distance between the online RSS and the offline RSS distribution. To further improve the accuracy of DFL in the case of multipath interference, Bocca et al. [3] used the moving average method, while Wang Jie adopted RSS differential measurement [15] to obtain RSS changes.

For the research object of weight models, Wilson et al. [16] proposed the Skew–Laplace weight model to characterize the mathematic relationship between the RSS attenuation and the target’s location. Yao Guo et al. [17] used the exponential Rayleigh model to depict the relationship between the RSS change and the position of the target. Kaltiokallio et al. [18] proposed a spatial weighting model to calculate the relationship between the target location and the RSS change in a multipath environment. Wang Jie et al. [19] proposed a saddle surface model to calculate the probability of different target positions in the localized area. Mager et al. [20] utilized an ellipsoid weight model to calculate the weight of each grid in the localized area. In order to satisfy the application requirements of RTI in roadside vehicle monitoring, Kassem et al. [9] used the support vector machine to monitor a roadside vehicle. Anderson et al. [3] realized roadside vehicle surveillance based on the improvement of the state-of-the-art weight mode of RTI. Recently, Jie [10] proposed a device-free vehicle speed estimation method based on the analysis of phases and the amplitude measurement of WiFi signals.

Referring to the previous works and the motivation discussed above, we develop a novel roadside vehicle 3D-DFL method. In this work, the real-time location and scale of a static vehicle in the monitoring WSN can be calculated automatically. It is important to note that this work is not a complete system as it can only be used for a static object. Nevertheless, the methods proposed in this paper, as a basis, are constructive and also available for the next steps of research (i.e., a moving vehicle monitoring system).

Our contributions in this paper are four-fold:

- We propose a screening method for blocked communication links based on the Gaussian kernel distance to differentiate whether a link is obscured or not by the vehicle in a cluttered environment.
- We propose the use of information entropy (IE) to evaluate the contribution of the obscured links; according to the contribution to the vehicle localization, different weights are assigned to each obscured link.
- We propose a combination weight allocation method based on the link weight and voxel spatial covariance to each voxel. Relying on the combination weight of each voxel, we generate a heat map of the monitoring area and estimate the location and the 3D information of the vehicle.
- We evaluate the proposed techniques with extensive experiments and analysis in a real testbed.

## 3. The Theory of 3D-DFL for Roadside Vehicle

In this section, we first describe the principle of DFL for a vehicle based on WSN, and then propose the 3D-DFL method for a roadside vehicle based on the weighted communication link and the grid method.

### 3.1. The Overview of DFL for a Vehicle Based on WSN

**Figure 1:**
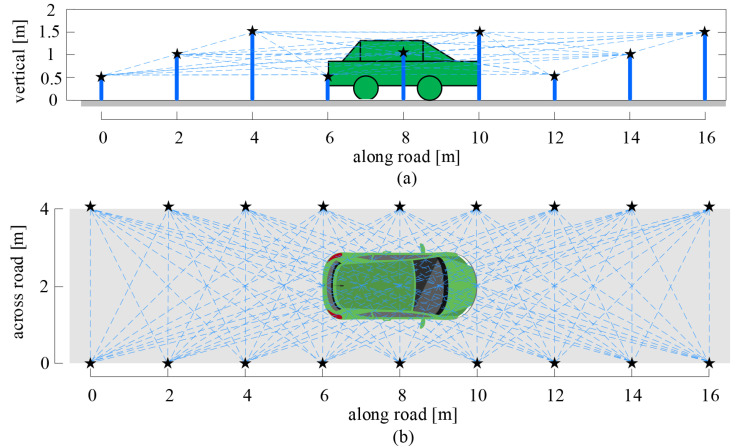
Diagram of the three-dimensional device-free localization (3D-DFL) model for a roadside vehicle. The gray shadow is the road, the black pentagrams are the sensor nodes, and the blue dashed lines are communication links. (**a**) Vertical view; (**b**) front view.

As shown in Figure 1, wireless sensor nodes are deployed in the monitoring area; the nodes communicate with each other to form a wireless sensor monitoring area, in which sensor nodes communicate with each other and form wireless communication links. When the vehicle appears in the monitoring area, the communication link is diffracted, reflected, scattered, and absorbed by the vehicle, that is, shadow fading occurs, which causes the received signal strength (RSS) of the communication link to change. In the outdoor environment, the wireless communication link rarely suffers from multipath interference, and so the main reason for the RSS attenuation is the shadow fading caused by the target. Thus, by observing RSS variation, we can judge whether a link is obscured by a vehicle or not. 

**Figure 2:**
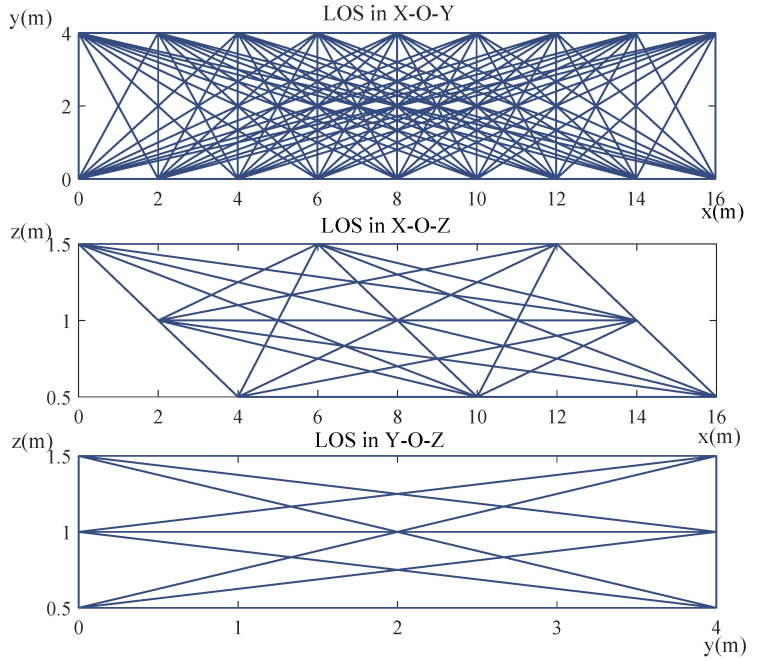
The projection density of wireless communication links on the X-O-Y plane, the X-O-Z plane, and the Y-O-Z plane, respectively.

As shown in Figure 1, for the 3D-DFL network for vehicle localization, we first set the direction along the road as the X axis, the direction across the road as the Y axis, and the direction vertical to the road as the Z axis. Then, the monitoring space is divided into voxels with a size of Δx×Δy×Δz (mm). In the 3D-DFL vehicle localization system, the 3D information of the vehicle is obtained according to the height, length, and width information of the vehicle. Therefore, the more accurate the estimation of the height, length, and width are, the more accurate the estimated 3D information of the vehicle is. In order to acquire a more accurate estimation for the vehicle, it is better to have more communication links to perform the measurement of the length, width and height of the vehicle. As shown in Figure 2, to decrease the calculation quantity, we project the communication links of the 3D wireless sensor monitoring network in the X-O-Y plane, the X-O-Z plane, and the Y-O-Z plane, respectively. Obviously, the performance of projection results involves the loss of 3D information. For example, the projection of links on the X-O-Y plane catches the information of length and width, with the loss of height information. This is similar for the X-O-Z plane and Y-O-Z plane; thus, when we calculate on one plane, we only concentrate on two of the three dimensions of information (e.g., length and width), and calculate the other dimensional information on one of other planes. Thus, we can calculate using two chosen planes to obtain the 3D characteristics. in this work, we choose the X-O-Y plane to estimate the length and width characteristics of the vehicle and estimate the height characteristic of the vehicle on the X-O-Z plane. Finally, the 3D characteristics of the vehicle can be obtained according to the length, width, and height of the vehicle from the X-O-Z plane and the X-O-Z plane, respectively.

### 3.2. The Voxels Partition and Its 2D Plane Projection

**Figure 3:**
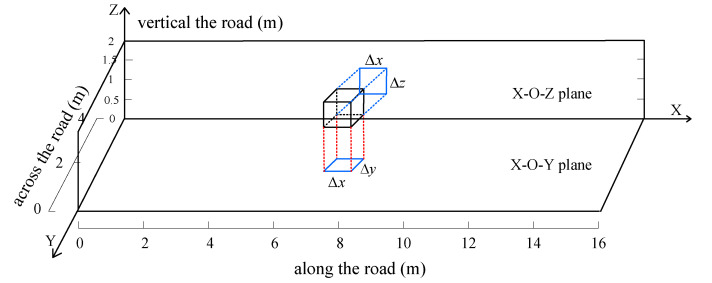
The projection diagram of one voxel on the X-O-Y plane and the X-O-Z plane.

In our 3D-DFL system, we divide the monitoring area into small voxels with a size of Δx×Δy×Δz, as shown in Figure 3. Then, the monitoring area can be formulated as a matrix V, which can be expressed mathematically as

(1) $$V=\left[v_{1},v_{2},\cdots ,v_{e},\cdots ,v_{E}\right]\;v_{i}=\left(x_{e},y_{e},z_{e}\right)$$

where $v_{e}$ denotes the $e^{th}$ voxel in V and $\left(x_{e},y_{e},z_{e}\right)$ is the 3D coordinate of the voxel $v_{e}$ with the origin coordinate in the lower left corner of the monitoring area.

Accordingly, the two-dimensional (2D) projection matrixes of the voxel V on the plane X-O-Y and the X-O-Z are $V_{XOY}$ and $V_{XOZ}$; specifically, they can be formulated as

(2) $$V_{XOY}=\left[v_{XOY}^{1},v_{XOY}^{2},\cdots ,v_{XOY}^{a},\cdots ,v_{XOY}^{r}\right]$$

(3) $$V_{XOZ}=\left[v_{XOZ}^{1},v_{XOZ}^{2},\cdots ,v_{XOZ}^{b},\cdots ,v_{XOZ}^{s}\right]$$

where $v_{XOY}^{a}$ is the $a^{th}$ 2D voxel in the plane of X-O-Y, *r* is the number of 2D voxels in the X-O-Y plane, $v_{XOZ}^{b}$ is the $b^{th}$ 2D voxel in the plane of X-O-Z, and *s* is the number of 2D voxels in the X-O-Z plane.

For example, if the size of the monitoring area is 16 m × 4 m × 1.5 m and the monitoring area is divided into small voxels with a size of 200 mm × 200 mm × 100 mm, we will have 80 voxels on the *x* axis, 20 voxels on the *y* axis, and 15 voxels on the *z* axis. Then, there are r = 80 × 20 voxels in the X-O-Y plane with a size of 200 mm × 200 mm and s = 80 × 15 voxels on the X-O-Z plane with a size of 200 mm × 100 mm.

### 3.3. The Procedure of This Work

**Figure 4:**
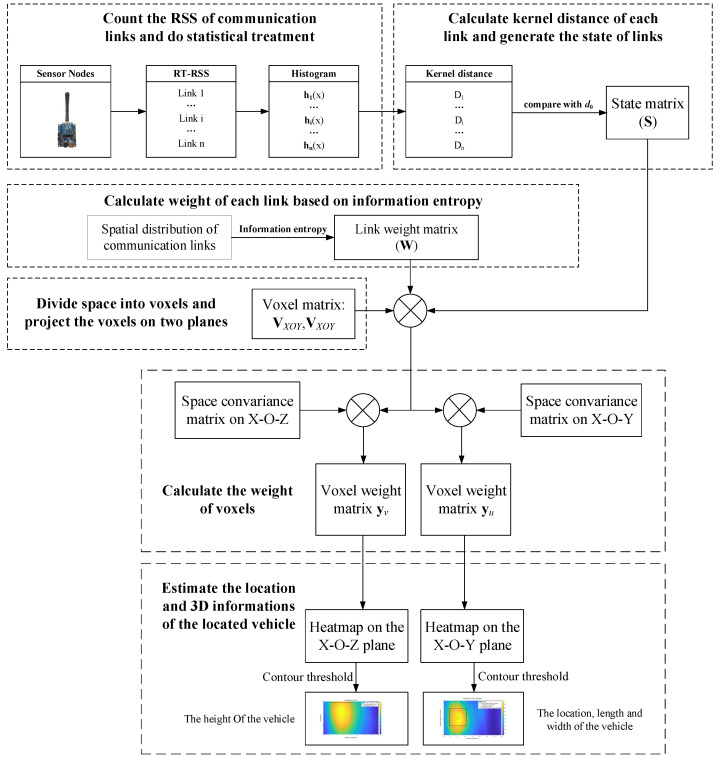
The flow chart of the 3D-DFL for a roadside vehicle.

As shown in Figure 4, the procedure of the 3D-DFL model for a roadside vehicle based on the combined weight of the communication link and the covariance of the voxels is as follows.

1. We count the real-time RSS (RT-RSS) values of communication links in a period of time and generate the online-phase RSS histograms as $h_{i}\left(x\right)$.
2. We calculate the kernel distance between the online-phase and offline-phase histograms of each communication link and compare the kernel distances with a threshold $d_{0}$; then, obtain the state (obscured or not obscured) of every communication link, expressed as a matrix S.
3. We utilize the information entropy principle to calculate the communication link weight matrix W, which is adopted to distinguish the contribution of the different obscured communication links to the 3D-DFL system.
4. We divide the monitored area into 3D voxels and project them on the X-O-Y plane and the X-O-Z plane, expressed as $V_{XOY}$ and $V_{XOZ}$.
5. We obtain the voxel weight matrix $y_{u}$ by multiplying the voxels’ space covariance of the X-O-Y plane, the matrix W, the matrix S and the voxel matrix $V_{XOY}$ in the same way, we can obtain the voxel weight matrix $y_{v}$ according to the product of the voxels’ space covariance in the X-O-Y plane, the matrix W, the matrix S and the voxel matrix $V_{XOZ}$.
6. We estimate the length and the width of the vehicle by taking an appropriate threshold for the heat map contours of the weight matrix $y_{u}$; in the same way, we can obtain the height of the vehicle from the weight matrix $y_{v}$.

## 4. The Selection Method for the Obscured Link

In order to clearly obtain the characteristic of the RSS variance and choose the links obscured by the vehicle, we first propose the use of a statistics method based on a histogram to represent the RSS distribution. Then, we use the Gaussian kernel distance to choose the obscured links.

### 4.1. RSS Characteristics Based on a Statistics Histogram

Let *K* be the number of sensor nodes in monitoring areas; thus, the number of communication links formed by any two nodes is K(K−1)/2. However, for the 3D-DFL system, only the communication links crossing the road can be used to monitor a vehicle in the sensor network. Supposing we have $M=\{m_{1},m_{2},\cdots ,m_{k},\cdots ,m_{K/2}\}$ and $N=\{n_{1},n_{2},\cdots ,n_{k},\cdots ,n_{K/2}\}$ sensor nodes on the two different sides of the road, respectively, $m_{i}$ and $n_{i}$ are the ID number of sensor nodes, where k=1,2,⋯,K/2. Then, the effective communication links across the road can be described as $L=\{\left(m_{1},n_{1}\right),\left(m_{1},n_{2}\right),\cdots ,\left(m_{K/2},n_{K/2}\right)\}$ with number of $K^{2}/4$.

The RSS of the $i^{th}$ communication link in *L* can be described as [2]

(4) $$R_{i}\left(x,t\right)=S_{i}-F_{i}\left(x,t\right)-D_{i}-f_{i}\left(t\right)$$

where $S_{i}$ is the transmitted signal strength, $F_{i}\left(x,t\right)$ is the signal strength attenuation caused by vehicle shadow fading, $D_{i}$ is the path transmission loss due to the transmission distance, and $f_{i}\left(t\right)$ is the measurement noise and the environmental interference to the RSS.

The RSS variance of the $i^{th}$ link at a different time can be written as

(5) $$\begin{array}{cc} \Delta R_{i} & =R_{i}\left(x,t_{2}\right)-R_{i}\left(x,t_{1}\right)\\ \; & =F_{i}\left(x,t_{2}\right)-F_{i}\left(x,t_{1}\right)-\left(f_{i}\left(t_{1}\right)-f_{i}\left(t_{2}\right)\right) \end{array}$$

Clearly, we can see that $\Delta R_{i}$ has a close relationship with shadowing attenuation $F_{i}\left(x,t\right)$ and $f_{i}\left(t\right)$. In order to accurately obtain the statistical characteristics of the variance RSS (e.g., mean, variance, etc.) and consider both $F_{i}\left(x,t\right)$ and $f_{i}\left(t\right)$ to determine the influence of the variance of RSS, we utilize a histogram to depict the distribution of the RSS of the link *i*, when the vehicle is in the monitoring area. Mathematically, we use $h_{i}\left(x\right)\in R^{2\times \eta }$ to mark the RSS variance, where η is the number of RSS values of the link *i* that appear during time $t_{2}-t_{1}$. The first column and the second column of $h_{i}\left(x\right)$ are the list of RSS values and the frequency of their corresponding RSS values, respectively. Specifically, they can be expressed as

(6) $${\left[h_{i}\left(x\right)\right]}_{1,j}=R_{j}$$

(7) $${\left[h_{i}\left(x\right)\right]}_{2,j}=p_{j}$$

In the 3D-DFL system, we propose the use of the kernel distance between the online and the offline RSS distribution of link *i* to determine whether the link is obscured. The offline phase means that there is no vehicle in the monitoring area, which is denoted by $h_{i}\left(0\right)$. In contrast, the vehicle is in the monitoring area during the online phase.

### 4.2. The Selecting Method Based on Kernel Distance

The kernel distance [21] can be regarded as the generalized distance between two high-dimensional vectors. If P and Q are point sets in the ϵ-dimensional Euclid space $R^{\epsilon }$ and the θ-dimensional Euclid space $R^{\theta }$, respectively, the kernel distance between P and Q can be formulated as follows,

(8) $$D_{K}^{2}\left(P,Q\right)=k\left(P,P\right)+k\left(Q,Q\right)-2k\left(P,Q\right)$$

where k(•) is a cross-similarity function that measures the similarity between two weighted *n*-dimensional vectors. It is determined by the following formula,

(9) $$k\left(P,Q\right)=\sum_{p\in P}\sum_{q\in Q}w\left(p\right)K\left(p,q\right)w\left(q\right)$$

where w(p) and w(q) are the weights of *p* and *q*, respectively. In the statistical histogram, these are frequencies of *p* and *q*, respectively. K(•) is the similarity function; here, we choose the Gaussian kernel function, which is formulated as [5]

(10) $$K\left(p,q\right)=exp\left(-\frac{{\left(p-q\right)}^{2}}{\sigma_{k}^{2}}\right)$$

where $\sigma_{k}$ is the width parameter of Gaussian kernel. Then, Formula (9) can be rewritten in the matrix form:

(11) $$k\left(P,Q\right)=W(P)K{\left[W\left(Q\right)\right]}^{T}$$

where W(P) and W(Q) are the weight vectors of P and Q, respectively. $K\in R^{\epsilon \times \theta }$ is the kernel matrix, and it can be expressed as

(12) $${\left[K\right]}_{i,j}=K\left(p_{i},q_{j}\right),\;p_{i}\in P,q_{j}\in Q$$

For the 3D-DFL, after obtaining the RSS histogram of all communication links, we introduce the kernel distance to calculate the difference between the RSS histograms of the link *i* with/without the vehicle in the monitoring area:

(13) $$D_{i}=d\left(h_{i}\left(x\right),h_{i}\left(0\right)\right)$$

where $D_{i}$ is the kernel distance and d(•) is the mapping from matrices to a real number. For the link *i*, if the kernel distance is greater than a threshold $d_{0}$, we consider that the link is obscured; otherwise, the link is not obscured. The state vector of all communication links can be formulated as

(14) $$S={\left[s_{1},s_{2},\cdots ,s_{i},\cdots ,s_{n}\right]}^{T}$$

(15) $$s_{i}=\left\{\begin{array}{c} \;0\;D_{i}<d_{0}\\ \;1\;D_{i}>d_{0} \end{array}\right.$$

where $s_{i}$ is the status of the link *i*. $d_{0}$ is the threshold of the kernel distance, and we set $d_{0}=1$ in our 3D-DFL system.

For the aforementioned histogram description of the RSS from link *i*, during the online phase, when the vehicle is in the monitoring area, the first column of $h_{i}\left(x\right)$ is the data set $P_{i}$ and the second column is the weighted vector $W(P_{i})$. Similarly, during the offline phase, the first column of $h_{i}\left(0\right)$ is the data set $Q_{i}$ and the second column of $h_{i}\left(0\right)$ is the weighted vector $W(Q_{i})$.

According to Formula (11), the cross-similarity between $h_{i}\left(x\right)$ and $h_{i}\left(0\right)$ is

(16) $$k\left(P_{i},Q_{i}\right)=W\left(P_{i}\right)K{\left[W\left(Q_{i}\right)\right]}^{T}$$

The kernel distance between $h_{i}\left(x\right)$ and $h_{i}\left(0\right)$ is

(17) $$\begin{array}{cc} D_{k}^{2}\left(P_{i},Q_{i}\right) & =k\left(P_{i},P_{i}\right)+k\left(Q_{i},Q_{i}\right)-2k\left(P_{i},Q_{i}\right)\\ \; & =d\left(h_{i}\left(x\right),h_{i}\left(0\right)\right)=D_{i} \end{array}$$

**Figure 5:**
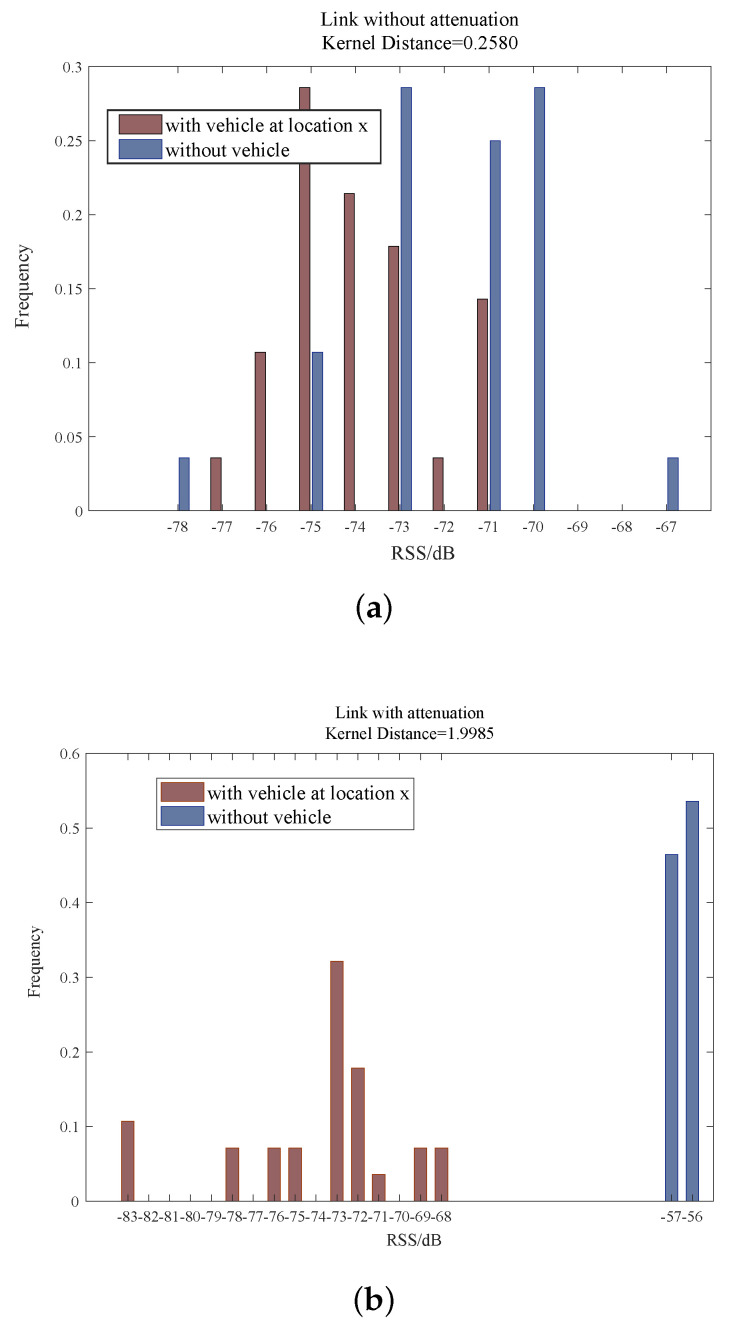
The validation of the selection method for the obscured link based on kernel distance. (**a**) The kernel distance of the unobscured link between the online phase and the offline phase. (**b**) The kernel distance of the obscured link between the online phase and the offline phase.

In order to validate the feasibility of the selection method for the obscured link based on the kernel distance, we analyze the obscured links and the unobscured links in detail by calculating the kernel distance between the online phase and the offline phase. Figure 5a shows the two RSS histograms of the unobscured link; they are obtained from the online phase and the offline phase, respectively. Clearly, for the unobscured link, although the frequencies of the RSS characteristics in the online phase are different from the offline phase, the value of the RSS characteristic in the online phase is almost the same as the offline phase (concentrated between −77 dB and −70 dB). Theoretically, for an unobscured link, the kernel distance between the online RSS distribution and the offline RSS distribution should be 0, but in fact it is 0.2580—this is because of the noise (described in Section 4.1), which introduces a frequency difference between the online phase and the offline phase. For the obscured link, as shown in Figure 5b, we can see that irrespective of the frequency or the RSS value, they both show an obvious difference due to the vehicle obstruction. Correspondingly, the kernel distance between the online and the offline RSS distribution is 1.9985. It can be seen that the difference between two RSS histograms can be extracted effectively by the kernel distance. If the threshold $d_{0}$ is set to 1, we can easily judge the link to be obscured by the vehicle, when its kernel distance is not less than 1.

## 5. The Location and Three-Dimensional Estimation of the Vehicle

In order to predict the 3D characteristics of the vehicle, we first assign weights to the communication links according to the information entropy principle. Second, we combine the weight of the communication link and the spatial correlation to obtain the weight of each voxel. Third, we use the voxel weight matrix to draw the heat map image and mark the contour lines in the heat map. Finally, we estimate the length, width, height and location of the vehicle according to the heat map image by taking ab appropriate threshold of the contour line.

### 5.1. The Weight Allocation Method Based on the Information Entropy Principle for the Obscured Links

**Figure 6:**
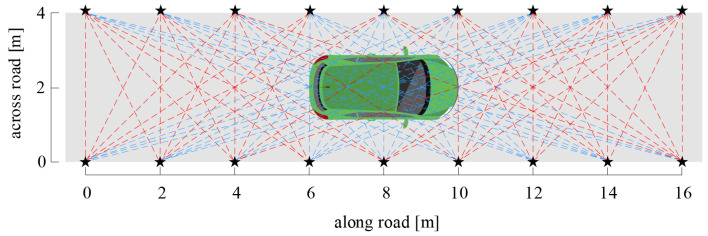
The projection of the 3D links in the X-O-Y plane. The red dashed lines denote the unobscured links; the light-blue dashed lines are the obscured links.

Assuming there are *n* communication links in the monitoring area, the location and the 3D information of the vehicle are contained in the communication links. However, the amount of information contained in each link is different for each link because of the difference in the spatial distribution. For example, Figure 6 shows the projection of the 3D links in the X-O-Y plane; the red dashed lines denote the unobscured links and the light-blue dashed lines are the obscured links. Obviously, we cannot extract the location and the 3D information of the vehicle from the red unobscured links, because the unobscured links have almost no RSS variance. For the light-blue links, although they contain the location and the 3D information according to their RSS variance, the amount of the information contained in them is different due to the difference in the spatial distribution. Here, we introduce an information entropy (IE) [22] method to calculate the contribution of different links to the location and the 3D information of the vehicle.

For the IE method, we take the change of a link from the unobscured state to the obscured state as a message, and the amount of the information contained in the message is closely related to the probability of the occurrence of the message. The smaller the probability of the message appearing, the greater the amount of information contained in the message. If the information contained in the message is represented by *I*, the IE of the link can be expressed by

(18) $$I=\log_{a}\frac{1}{P(x)}$$

where the unit of IE is related to *a*. P(x) is the probability that the link is obscured, as the link only has two states (e.g., obscured or unobscured); thus, we assign *a* to be 2.

**Figure 7:**
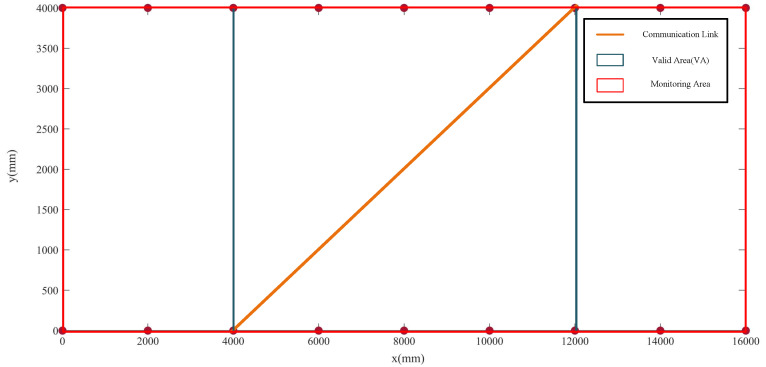
Valid area of the communication link.

As shown in Figure 7, to calculate the probability P(x) of a link being obscured, we define the valid area (VA) of the link as a rectangle whose diagonal is the link, and the edges are parallel to the coordinate axis of the projection plane. We use the VA to approximate the area in which the link can monitored; in other words, the link can effectively detect whether or not there is an obstacle in the VA. We define the VA of link *i* as $A_{i}$ and the total area of the projection plane as *A*; then, the probability of link *i* being obscured is

(19) $$P_{i}=\frac{A_{i}}{A}$$

Note that the VA is not the real area, meaning that the link can be obscured, and the $P_{i}$ is also not the real probability of the link being obscured. The definitions are used to evaluate the IE of communication links. According to Formulas (18) and (19), the IE of link *i* is

(20) $$I_{i}=\log_{2}\frac{1}{P_{i}}$$

**Figure 8:**
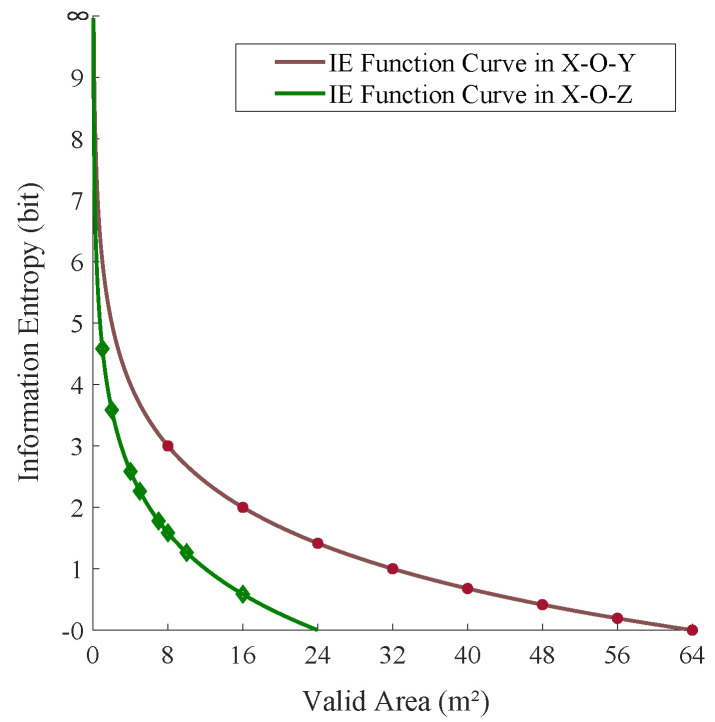
The relationship between the valid area (VA) and the information entropy (IE) for the link in the X-O-Y and X-O-Z planes, respectively.

**Figure 9:**
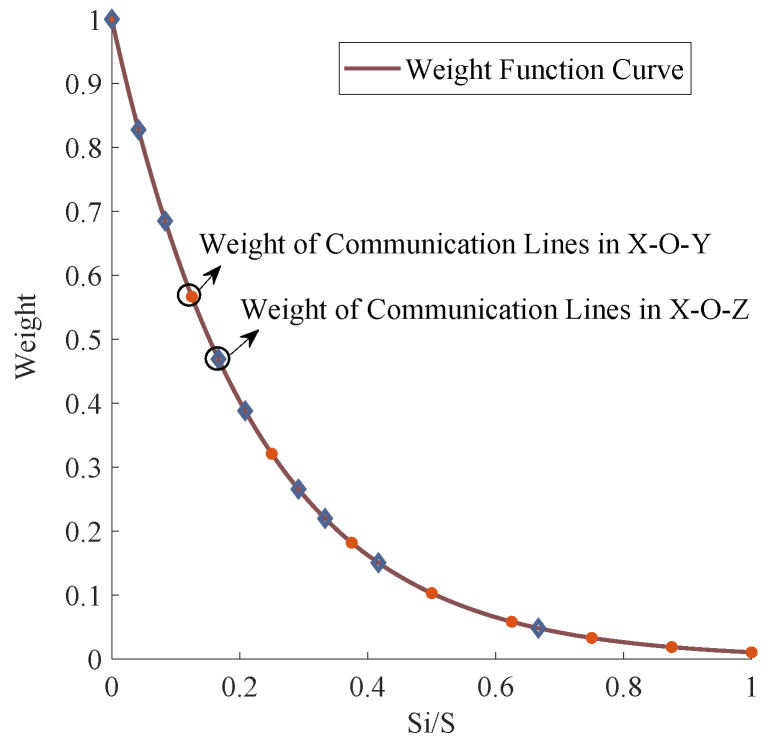
The relationship between the weight and the $S_{i}/S$ of link *i* in the X-O-Y and the X-O-Z planes, according to the exponential decay function.

Figure 8 intuitively demonstrates the relationship between the VA and the IE of link *i*. Obviously, the smaller the VA of the link, the higher the IE. Correspondingly, we assign a higher weight to the link with a smaller VA; this is because that the link with a smaller VA has a higher IE and contributes more information to the localization and 3D size of the vehicle. Considering the extreme case that the VA of the link is 0, when the link is perpendicular to the road, the probability $P_{i}$ of the link is 0, according to Formula (19), which means the IE of the link is *∞* according to Formula (20), and this phenomenon will result in the other links having no weight. To avoid such problem and consider the IE distribution curve feature at the same time, we propose a new exponential decay function to assign weights for all the obscured links, as shown in Figure 9. Mathematically, this is expressed as

(21) $$w_{i}=exp\left(-\frac{{\left(A_{i}/A\right)}^{\alpha }}{\sigma_{l}}\right)$$

where α and $\sigma_{l}$ are the customized parameters, $S_{i}$ is the VA of the link *i* in the projection plane, and *S* is the total area of the projection plane.

According to Formula (21), the weight matrix of each communication link can be obtained:

(22) $$W={\left[w_{1},w_{2},\cdots ,w_{i},\cdots ,w_{n}\right]}^{T}$$

where $w_{i}$ is the weight of the communication link *i*.

### 5.2. The Space Covariance of Voxels

The voxels occupied by the vehicle are physically continuous in the spatial monitoring area; i.e., the contribution of a voxel and its surrounding voxels to the location and the 3D information of the vehicle are close to each other. The closer the distance between each voxel, the greater the correlation, and vice versa. Here, we propose the use of a random spatial covariance model [2] to specify the correlation between each voxel. The covariance matrix C can be given by

(23) $$\left[C_{a,b}\right]=\sigma_{i}^{2}e^{-d_{a,b}/\sigma_{c}}$$

where $d_{a,b}$ is the Euclidean distance between the two voxel centers: $\sigma_{c}$ is the spatial constant and $\sigma_{i}$ is the variance in each voxel. $C\in R^{r\times r}$ in the X-O-Y plane and $C\in R^{s\times s}$ in the X-O-Z plane.

**Figure 10:**
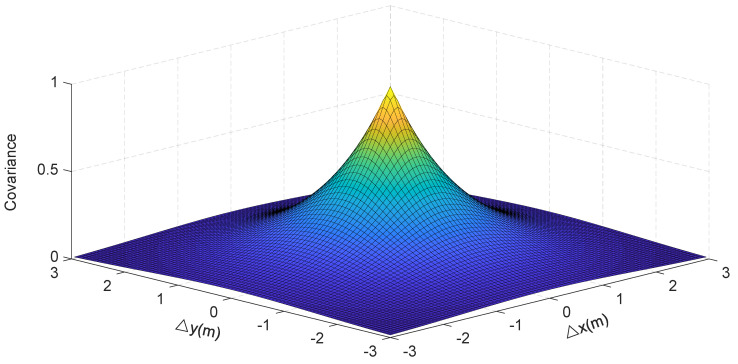
The relationship between the distance and the covariance of different voxels.

Applying this model, the correlations between each voxel are shown in Figure 10. We can see from the figure that with the increase of the *x* distance and *y* distance between every two voxels, their correlation decreases gradually.

### 5.3. The Location and the 3D Information of the Vehicle

#### 5.3.1. The Voxel Weight Based on the Link Weight and the Spatial Covariance

The weight of each voxel is determined by the weight of the link passing through the voxel and the spatial covariance. We call the weight caused by the link passing through a voxel the native-weight and call the weight obtained by the spatial covariance the derivative-weight. The weight of the voxel is the sum of the native weight and the derivative weight. If there are several links passing through one voxel, the native weight of the voxel is the sum of the weight of the several links passing through it, and the final weight of the voxel equals the sum of the native-weight and the derivative-weight.

The weight matrix of the voxels in the X-O-Y plane is

(24) $$y_{u}=C_{u}\left(\sum_{i}^{n}s_{i}u_{i}\right)$$

The weight matrix of the voxels in the X-O-Z plane is

(25) $$y_{v}=C_{v}\left(\sum_{i}^{n}s_{i}v_{i}\right)$$

where *n* is the number of communication links, $s_{i}$ is the status of link *i*, and $u_{i}$ is the sum of the voxel weights generated by the links passing through it in the X-O-Y plane (native-weight), which is a *r*-dimensional vector. $v_{i}$ is the sum of the voxel weights generated by the links passing through it in the X-O-Z plane; it is a *s*-dimensional vector. $C_{u}$ and $C_{v}$ are the projections of the spatial covariance matrix C in X-O-Y and X-O-Z planes, respectively. $C_{u}$ is a *r*-dimensional square matrix and $C_{v}$ is a *s*-dimensional square matrix. $y_{u}$ and $y_{v}$ represent the weight of each voxel in the X-O-Y plane and the X-O-Z plane, respectively. Mathematically, $u_{i}$ and $v_{i}$ can be expressed as

(26) $$u_{i}=w_{i}S_{u}^{i}⊙V_{XOY}$$

(27) $$v_{i}=w_{i}S_{v}^{i}⊙V_{XOZ}$$

where $w_{i}$ is the weight of link *i*. $S_{u}^{i}$ and $S_{v}^{i}$ are the voxel selection matrixes; they are generated by link *i*. Their elements have a one-to-one correspondence with voxels in their respective plane. When a voxel is passed through by link *i*, the corresponding element value is 1; otherwise, the value is 0. $V_{XOY}$ and $V_{XOZ}$ are the voxel matrixes, and all their elements equal 1; $V_{XOY}$ is a *r*-dimensional vector and $V_{XOZ}$ is a *s*-dimensional vector. *r* and *s* are the number of voxels in X-O-Y and Y-O-Z planes, respectively. ⊙ indicates the Hadamard multiplication.

#### 5.3.2. The Location and the 3D Information Estimation Based on Contour Threshold

First, we obtain the heat map images according to the weight matrix of the voxels $y_{u}$ and $y_{v}$ in the X-O-Y plane and X-O-Z plane, respectively. From the aforementioned analysis, we know that the width and the length of the vehicle are contained in the heat map image of $y_{u}$, while the height information is contained the heat map image of $y_{v}$. Here, we propose the use of the contour threshold to capture the location and the 3D information from the heat map image of $y_{u}$ and $y_{v}$. The specific procedure of the location and the 3D information estimation are as follows:

We obtain the contour threshold by normalizing the voxel matrix $y_{u}$ and $y_{v}$ during the offline training phase, in which the contour threshold of the length, width and height of the vehicle can be calibrated according to the actual 3D information of the vehicle. The normalized contour is a relative value; thus, even if the vehicle size and location change, the threshold value of the contour is still available. For example, in the X-O-Y plane, we obtain the contour threshold in the length, width, and height directions by the offline training when the vehicle is at the location of (8 m, 2 m), and then take the same contour threshold when the vehicle in other locations. In this way, we can roughly estimate the length and the width of the vehicle and then obtain the center of gravity in the threshold, regarding the center as the location of the vehicle. In the same way, we can obtain the height of the vehicle in the X-O-Z plane.

## 6. Experiments

### 6.1. Experimental Setup

**Figure 11:**
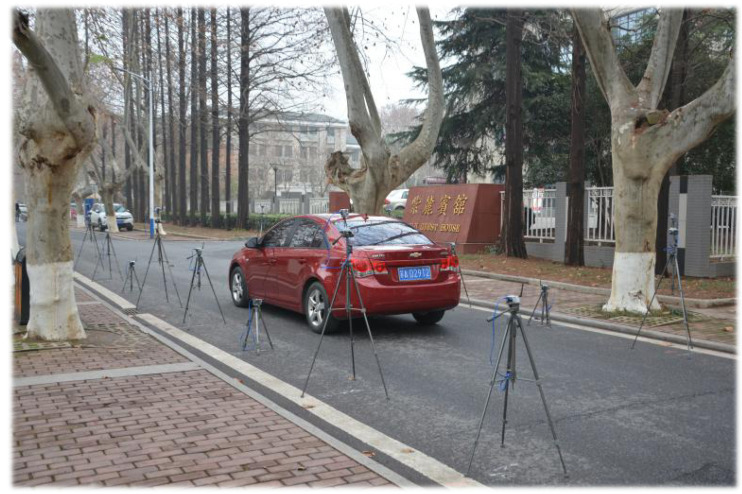
The layout of the experiment.

We build the 3D-DFL field experiment testbed according to the deployment of Figure 1. A total of 18 nodes are evenly distributed on both sides of the road. The distance between the two sides is 4 m. Sensor nodes are placed on the same side with a spacing of 2 m, and the nodes are fixed at three different heights of 1.5 m, 1 m, and 0.5 m, as shown in Figure 11.

We adopt CC2530 sensor nodes operating at 2.4 GHz. Each node has its own ID number and a communication channel list—channel 12, channel 14, channel 22, and channel 26—which are programmed in advance. All sensor nodes run the multi-spin [23] protocol and work at the maximum power (4.5 dBm). Specifically, the multi-spin protocol makes all sensor nodes take turns to transmit data according to their node IDs. At any particular time, only one node sends data, while the other nodes receive data. When a sensor node fails or a packet is dropped, a timer will fire to ensure that subsequent sensor nodes continue to send data. All sensor nodes simultaneously switch to the next communication channel, which is defined in the channel list, after the node with the largest ID number finishes sending data. If the current operating channel is the last one in the channel list (e.g., channel 26), then a multi-spin protocol will choose the first channel to move on. The 3D-DFL system selects the RSS of the wireless channel with the smallest packet loss rate to localize the vehicle. In addition, a based station node is deployed outside the monitoring area to receive data from all the communication links and forward them to a laptop via a USB interface.

The time taken in one loop of sequential measurement is 72 μs, meaning a 2 mm movement of the vehicle with a speed of 100 km/h during one measurement process. The voxel we divided, with a scale of 200 mm in the direction of the vehicle’s speed, requires the movement distance of the vehicle to be within 20 mm in one measurement process to satisfy the Nyquist spatial sampling criterion (the limitation of the vehicle speed is related to the scale of the voxel). Thus, this system satisfies the requirements of moving vehicle measurement.

### 6.2. Results and Discussion

During the experiment, we parked the vehicle at the location of (0 m, 2 m), (4 m, 2 m), (8 m, 2 m), (12 m, 2 m), and (16 m, 2 m), respectively. The Gaussian kernel width $\sigma_{k}$ for the obscured link selection, the custom parameter α and $\sigma_{l}$, the variance of each voxel $\sigma_{i}$ in the weight allocation method based on IE, and the space constant $\sigma_{c}$ in the covariance model are described in Table 1.

We utilized the root mean square error (RMSE) [2] to estimate the accuracy of the localization of 3D-DFL. As shown in Table 2, the average localization accuracy of our 3D-DFL was 0.808 m. The estimation accuracies of the 3D-DFL system were 1.820 m, 0.247 m, 0.250 m, 0.255 m, and 1.466 m, respectively. Clearly, compared with the scenarios in which the vehicle is located at the edge of the sensor network, we can obtain more accurate results (e.g., 0.247 m, 0.250 m, and 0.255 m) when the vehicle is completely in the monitoring area. This is because of the lower link density at the edge of the network (e.g., the locations of (0 m, 2 m) and (16 m, 2 m)).

**Figure 12:**
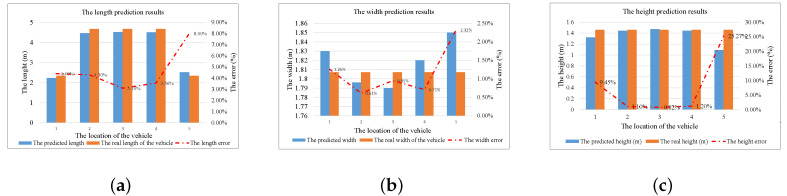
The estimation of the 3D information. (**a**) The length prediction results of the vehicle. (**b**) The width prediction results of the vehicle. (**c**) The height prediction results of the vehicle.

In addition, our 3D-DFL system can also accurately estimate the 3D information of the vehicle. The real size of the vehicle used in our experiment is 4.667 m × 1.807 m × 1.460 m. Figure 12a shows the relationship between the real length of the vehicle and the estimated length. Due to only half part of the vehicle entering the monitoring area, at the first and fifth locations, the real length is 2.33 m. The 3D-DFL method can excellently estimate the length of the vehicle with average error of 3.65%, when the vehicle is totally in the monitoring area. Furthermore, the length estimation results follow the same trend as the localization results, showing that we can obtain better length estimation results when the vehicle is in the middle of the monitoring area compared with the results from the edge of the monitoring area, with an almost 41.05% improvement. For the width and the height estimation results, we also emphasize the scenarios in which the vehicle is completely within the monitoring area. As shown in Figure 12b,c, we can see that the 3D-DFL method can obtain about a 0.75% average width error and a 1.04% average height error. Considering the real size of the vehicle, in terms of length, width and height, our 3D-DFL method can estimate the 3D information of the vehicle with errors of 0.17 m, 0.01 m, and 0.015 m, respectively.

**Figure 13:**
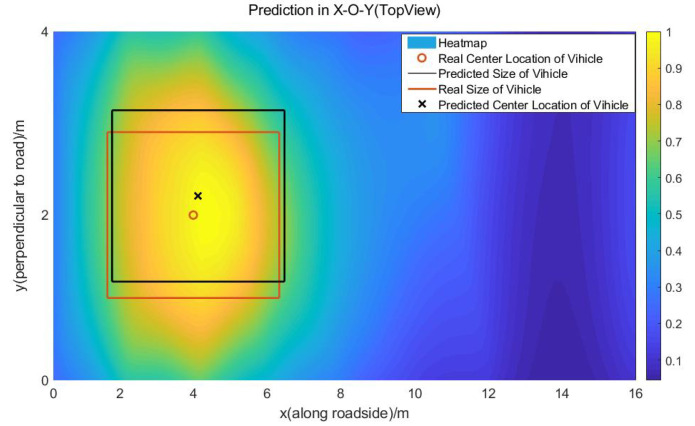
The heat map image of the vehicle at the location of (4 m, 2 m) in the X-O-Y plane. The central position, the length, and the width of the vehicle are predicted with a length contour threshold of 0.7 and width threshold of 0.9.

**Figure 14:**
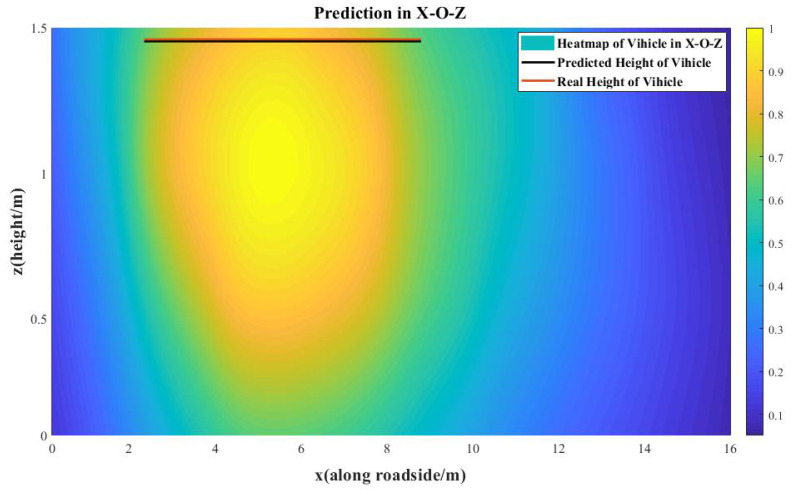
The heat map image of the vehicle at the location of (4 m, 2 m) in the X-O-Z plane. The height of the vehicle is predicted with a height contour threshold of 0.85.

In order to further explain the method of the localization and 3D information estimation based on the contour threshold, we analyzed the heat map image of the vehicle, when it was located at the coordinates of (4 m, 2 m) in the X-O-Y plane, as shown in Figure 13. In the heat map image, the density of the contour lines was 0.05. As the vehicle is approximately a rectangle in the X-O-Y plane, we took the vehicle boundary as four straight lines parallel with the *x* axis and *y* axis tangent to the threshold contour. According to the offline calibration, we selected the length contour threshold as 0.7, when the length of contour was the closest to the vehicle’s length. In the same way, we obtained the width contour threshold as 0.9. On the X-O-Z plane, we only needed to obtain the height of the vehicle. Thus, we took the boundary of the vehicle as a single straight line parallel with the *x* axis tangent to the threshold contour to represent the height of the vehicle. According to the offline calibration, we took the height contour threshold as 0.85. As shown in Figure 14, because the height of the sensor network is approximately equal to the height of the vehicle, it is easy to see that the predicted height equals the maximum height of the sensor network. At the top edge of the X-O-Z plane, there are few links, so the error is obviously larger than the central part of the X-O-Z plane. To improve the accuracy of height prediction, the height range of the sensor network should be increased, thus increasing the maximum height of nodes.

### 6.3. The Influence of Each Parameter to the Error of the Prediction Results

#### 6.3.1. Effect of Gaussian Kernel Width Parameter $\sigma_{k}$ on Link Screening Results

**Figure 15:**
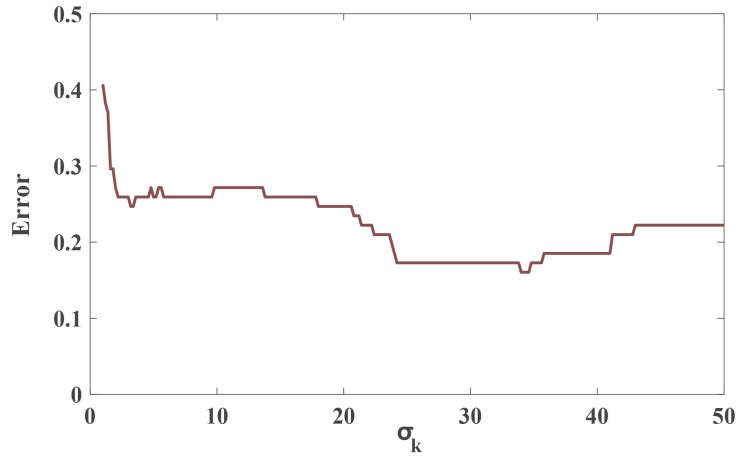
The relationship between the error rate in which the obscured links are wrongly selected as unobscured ones with changing $\sigma_{k}$ values.

In our experiments, measurement noise or environmental interference always result in the fluctuation of the RSS value, causing us to easily make a mistake when determining whether a link is obscured. As shown in Formula (10), we know that $\sigma_{k}$ can be analogous to a scale to compare the similarity between two different data sets, that is, it impacts the sensitivity of differentiating between two different data sets. In other words, a smaller value of $\sigma_{k}$ is good for restraining the measurement noise. However, it is less sensitive to differentiating the two data sets. As a result, the value of $\sigma_{k}$ has a great impact on the accuracy of selecting the obscured links. Figure 15 shows the error rate in which the obscured links are wrongly selected as unobscured ones with changing $\sigma_{k}$ values. We can see that the error rate is lower when the value of $\sigma_{k}$ is between 25 and 35. Thus, during our experiments, we chose $\sigma_{k}$ as 30.

#### 6.3.2. The Influence of α and $\sigma_{l}$ to the Weight Allocation Method Based on the IE Method

**Figure 16:**
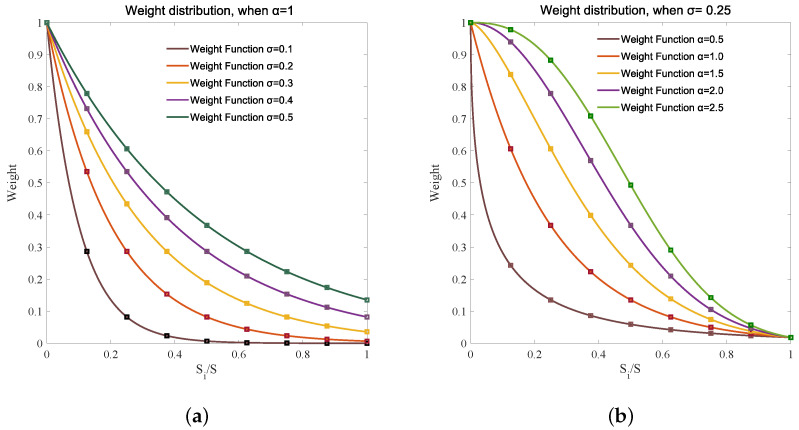
Different weight function curves with different parameters of weight function. (**a**) The distribution characteristics of the obscured link weight with different $\sigma_{l}$ values. (**b**) The characteristics of the obscured link weight distribution with different α values.

According to Formula (21), the weight allocation method for the obscured link is determined by the parameters α and $\sigma_{l}$, which are independent of each other. An excessive α would polarize link weights, leading to the distortion of information contained in the obscured links. $\sigma_{l}$ determines the distribution value of the obscured link weight as a whole. The larger the $\sigma_{l}$ is, the greater the overall distribution, and vice versa. Specifically, Figure 16a shows the distribution characteristics of the obscured link weight with different $\sigma_{l}$. The link weight distribution is relatively consistent with the principle of weight distribution when α is 1 and $\sigma_{l}$ is between 0.2 and 0.3. The overall weight distribution of the brown curve ($\sigma_{l}$ = 0.1) is too small and the overall weight distributions of the purple ($\sigma_{l}$ = 0.4) and green ($\sigma_{l}$ = 0.5) curves are larger.

Figure 16b shows the characteristics of the obscured link weight distribution with different α values. The link weight distribution is reasonable when $\sigma_{l}$ = 0.25 and α is between 1 and 1.5, because the shape of the curve and the link weight distribution in this scale is similar to the IE curve. The weight distribution of the brown curve (α = 0.5) is too dense, and except for the point at which the weight is 1, the other weights are too small; that is, polarization appears. Regarding the weight distribution of the purple (α = 2) and the green (α = 2.5) curves, the large weight points are too close.

**Figure 17:**
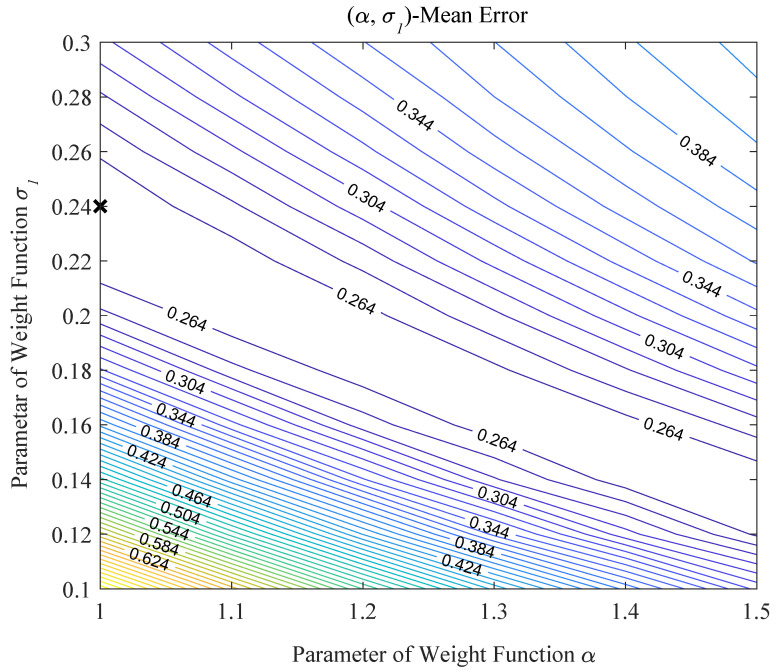
The relationship between the average localization errors and the two parameters α and $\sigma_{l}$. The x axis is parameter α and the y axis is parameter $\sigma_{l}$. The contour lines are the average localization error. The black cross is the local minimum error.

According to the aforementioned analysis, we further explore the quantitative relationship between the average localization errors and the two parameters (e.g., α and $\sigma_{l}$). As shown in Figure 17, the x axis is the value of α, the y axis is the value of $\sigma_{l}$ and the contours denote the localization errors. Referring to the diagram, we can see that there are innumerable points that lead to the average localization being smallest. Thus, we take a normal value as the local minimum average localization error point, where α is 1 and $\sigma_{l}$ is 0.24.

### 6.4. The Influence of the Sensor Network Topology on the Localization Accuracy

Figure 2 shows the density distribution of the communication links in the 3D-DFL network for the vehicle. We find that the link distribution is not uniform in the sensor network. The density of the communication links is smaller at the edge of the monitoring area than in the middle of the monitoring area. The 3D-DFL technique uses the RSS variance of communication links to obtain the location and the 3D information of the vehicle. Therefore, a lower density of communication links results in the reduction of localizing accuracy at the edge of the network being inevitable, which has been proved in Table 2. To improve the location and the 3D information estimation, we can change the topology of sensor network nodes and increase the communication link density at the edge of the monitoring area.

## 7. Conclusions

In this paper, we develop a 3D-DFL method for roadside vehicles. On the one hand, we propose a novel method of communication link weight allocation based on IE. In combination with other methods, we realize the real-time localization and 3D-scale estimation of a static vehicle. Experiments show that the IE-based communication link weight distribution method can achieve good vehicle localization and 3D information estimation. On the other hand, the work we have done is aimed at a static object and is therefore not a complete work. Additionally, there are some theories which are not rigorous, such as the reasoning behind the projections chosen and the definition of VA, et al. Nevertheless, our work is also constructive as a basic theory for follow-up, as the theories in this paper are still available for use in moving vehicle surveillance. In our future work, we will focus on 3D-DFL surveillance for a dynamic vehicle and simultaneously optimize the methods in our previous works.

## Figures and Tables

**Table 1:** Parameters used in the experiment.

Parameter	Value	Description
$\sigma_{k}$	30	Gaussian kernel width
α	1	Customized parameter in the IE method
$\sigma_{l}$	0.24	Customized parameter in the IE method
$\sigma_{i}$	1	Variance of each voxel in the covariance model
$\sigma_{c}$	0.9	Space constant of the covariance model

**Table 2:** The localization results in the X-O-Y plane. RMSE: root mean square error.

ID	Real Coordinates of the Vehicle (m)	Predicted Coordinates of the Vehicle (m)	RMSE (m)
1	(0, 2)	(1.80, 2.27)	1.820
2	(4, 2)	(3.94, 2.24)	0.247
3	(8, 2)	(7.99, 2.25)	0.250
4	(12, 2)	(11.89, 2.23)	0.255
5	(16, 2)	(14.57, 2.33)	1.468
Average	–	–	0.808

## References

[1] Wilson J., Patwari N. (2010). Radio tomographic imaging with wireless networks. IEEE Trans. Mob. Comput..

[2] Viani F., Rocca P., Oliveri G., Trinchero D., Massa A. (2011). Localization, tracking, and imaging of targets in wireless sensor networks: An invited review. Radio Sci..

[3] Zhao Y., Patwari N., Phillips J.M., Venkatasubramanian S. Radio tomographic imaging and tracking of stationary and moving people via Kernel Distance. Proceedings of the 2013 ACM/IEEE International Conference on Information Processing in Sensor Networks (IPSN).

[4] Zhang L., Gao Q., Ma X., Wang J., Yang T., Wang H. (2018). DeFi: Robust Training-Free Device-Free Wireless Localization with wiFi. IEEE Trans. Veh. Technol..

[5] Shi S., Sigg S., Chen L., Ji Y. (2018). Accurate Location Tracking From CSI-Based Passive Device-Free Probabilistic Fingerprinting. IEEE Trans. Veh. Technol..

[6] Konings D., Alam F., Noble F., Lai E.M. (2019). SpringLoc: A Device-Free Localization Technique for Indoor Positioning and Tracking Using Adaptive RSSI Spring Relaxation. IEEE Access.

[7] Zhang L., Wang H. (2019). Device-Free Tracking via Joint Velocity and AOA Estimation With Commodity WiFi. IEEE Sens. J..

[8] Anderson C.R., Martin R.K., Walker T.O., Thomas R.W. (2014). Radio Tomography for Roadside Surveillance. IEEE J. Sel. Top. Signal Process..

[9] Kassem N., Kosba A.E., Youssef M. RF-based vehicle detection and speed estimation. Proceedings of the 2012 IEEE 75th Vehicular Technology Conference (VTC Spring).

[10] Wang J., Tong J., Gao Q., Wu Z., Bi S., Wang H. (2018). Device-Free Vehicle Speed Estimation with WiFi. IEEE Trans. Veh. Technol..

[11] Youssef M., Mah M., Agrawala A. Challenges: Device-free passive localization for wireless environments. Proceedings of the 13th Annual ACM International Conference on Mobile Computing and Networking.

[12] Moussa M., Youssef M. Smart devices for smart environments: Device-free passive detection in real environments. Proceedings of the 2009 IEEE International Conference on Pervasive Computing and Communications.

[13] Wilson J., Patwari N. (2011). See-through walls: Motion tracking using variance-based radio tomography networks. IEEE Trans. Mob. Comput..

[14] Bocca M., Kaltiokallio O., Patwari N. Radio tomographic imaging for ambient assisted living. Proceedings of the 2013 ACM/IEEE International Conference on Information Processing in Sensor Networks (IPSN).

[15] Wang J., Gao Q., Wang H., Cheng P., Xin K. (2015). Device-free localization with multidimensional wireless link information. IEEE Trans. Veh. Technol..

[16] Wilson J., Patwari N. (2012). A fade-level skew-laplace signal strength model for device-free localization with wireless networks. IEEE Trans. Mob. Comput..

[17] Guo Y., Huang K., Jiang N., Guo X., Li Y., Wang G. (2015). An exponential-Rayleigh model for RSS-based device-free localization and tracking. IEEE Trans. Mob. Comput..

[18] Kaltiokallio O., Bocca M., Patwari N. (2014). A fade level-based spatial model for radio tomographic imaging. IEEE Trans. Mob. Comput..

[19] Wang J., Gao Q., Pan M., Zhang X., Yu Y., Wang H. (2016). Toward Accurate Device-Free Wireless Localization With a Saddle Surface Model. IEEE Trans. Veh. Technol..

[20] Mager B., Patwari N., Bocca M. Fall detection using RF sensor networks. Proceedings of the 2013 IEEE 24th Annual International Symposium on Personal, Indoor, and Mobile Radio Communications (PIMRC).

[21] Phillips J.M., Venkatasubramanian S. (2011). A gentle introduction to the kernel distance. arXiv.

[22] Nuez J.A., Cincotta P.M., Wachlin F.C. (1996). Information entropy. Celest. Mech. Dyn. Astron..

[23] Kaltiokallio O., Bocca M., Patwari N. Enhancing the accuracy of radio tomographic imaging using channel diversity. Proceedings of the 2012 IEEE 9th International Conference on Mobile Ad-Hoc and Sensor Systems (MASS 2012).

